# The effect of distraction versus post-event processing on cortisol recovery in individuals with elevated social anxiety

**DOI:** 10.1016/j.cpnec.2022.100142

**Published:** 2022-05-13

**Authors:** Shunta Maeda, Chihiro Moriishi, Hiroyoshi Ogishima, Hironori Shimada

**Affiliations:** a Graduate School of Education, Tohoku University, Miyagi, Japan; b Graduate School of Human Sciences, Waseda University, Saitama, Japan; c Research Institute for Future Design, Kochi University of Technology, Kochi, Japan; d Faculty of Human Sciences, Waseda University, Saitama, Japan

**Keywords:** Distraction, Post-event processing, Rumination, Cortisol, Social anxiety

## Abstract

There are preliminary findings that repetitive thinking on social situations (post-event processing; PEP) is associated with impaired cortisol recovery after experiencing social evaluative stressors. However, no studies have examined the effect of experimental manipulation of PEP on cortisol recovery among socially anxious individuals. The aim of the present study was to examine the effect of distraction on cortisol recovery following a social-evaluative stressor in individuals with subclinical social anxiety symptoms. A total of 40 participants, who scored >30 on the Liebowitz Social Anxiety Scale, completed a standardized stress test (the Trier Social Stress Test; TSST). They were then randomized to complete either a 10-min distraction or PEP induction task. Subjective anxiety and salivary cortisol levels were assessed at −20, −10, 0, +10, +20, +30, +40, and +50 min, with respect to the TSST offset. Contrary to the hypothesis, no difference in cortisol recovery was observed between distraction induction and PEP induction. These findings suggest that short-term distraction induction may not be sufficient to promote cortisol recovery in individuals with elevated social anxiety.

## Highlights

• Effect of distraction on cortisol recovery was tested in individuals with elevated social anxiety.
• No effect of distraction on cortisol recovery in comparison to post-event processing induction.
• Short-term distraction induction may not be sufficient for individuals with social anxiety to promote cortisol recovery.

## 1 Introduction

Social anxiety is characterized by marked fear of being scrutinized during social interactions [1]. Regarding the pathophysiology of social anxiety, the role of the hypothalamic-pituitary-adrenal (HPA) axis has been assumed to be substantial. The HPA axis regulates the release of cortisol, which is an important hormone associated with psychological, physiological, and physical health functioning. While the HPA axis is reactive to various kinds of stressors including emotional and physical ones, stressors of an uncontrollable and social-evaluative nature are especially associated with cortisol release [2]. As one of the core features of social anxiety is fear of being evaluated, HPA axis responses to psychosocial stressors have been intensively investigated [[3], [4], [5], [6]]. Although the directionality of alteration in HPA axis responsiveness in social anxiety is not necessarily consistent in existing findings, a recent meta-analysis revealed that individuals with social anxiety disorder (SAD) show heightened cortisol responses to psychological stressors, and this effect is most prominent during recovery periods (more than 25 min post-stressor offset [7]). Additionally, previous findings suggest that cortisol responses can facilitate the avoidance of socially threatening stimuli among individuals with SAD [8,9]. Such avoidance behaviors can prevent individuals from habituating to socially threatening situations, in turn, leading to persistent fear responses. Thus, it is important to develop a strategy to facilitate cortisol recovery in individuals with social anxiety.

In past studies, researchers attempted to understand the mechanism underlying individual differences in cortisol recovery in terms of perseverative cognition. That is, perseverative cognition in response to stressors has been hypothesized to prolong not only emotional responses but also physiological responses. In particular, rumination has been found to be associated with impaired cortisol recovery [10]. The perseverative cognition hypothesis may provide a plausible explanation for impaired cortisol recovery in individuals with social anxiety. Indeed, cognitive models of social anxiety emphasize the importance of repetitive thinking about social situations after leaving or escaping them [11]. This repetitive thinking pattern is called post-event processing (PEP, hereafter), and there is ample evidence indicating that social anxiety predicts the occurrence of PEP [[12], [13], [14]]. Following this line of reasoning, it is possible that PEP can account for impaired cortisol recovery in individuals with social anxiety, by causing prolonged processing of social-evaluative threat. There have been some preliminary attempts to examine the effect of PEP on impaired cortisol recovery. Maeda et al. [15] examined whether social anxiety and post-event processing of social-evaluative stressors predict impaired cortisol recovery in unselected non-clinical populations. Their findings indicated that PEP predicts impaired cortisol recovery for those with low levels of social anxiety but not for those with high levels of social anxiety. These findings almost contradicted the initial hypothesis, and the necessity for further research with socially anxious populations was emphasized. A more recent study [16] investigated the relationship between trait emotion regulation strategies (worry, rumination, and reappraisal) and cortisol recovery following social-evaluative stressors in both individuals with diagnosed SAD and healthy controls. In this study, rumination was defined as the thoughts on possible causes and consequences of their depressed mood, which is different but relevant to PEP. Their findings indicated that trait worry and rumination were associated with impaired cortisol recovery in the whole sample.

If the impaired cortisol recovery in SAD populations could be explained by PEP, then engaging in adaptive emotion regulation strategies that counteract PEP may promote cortisol recovery. Lewis et al. [16] examined the effect of trait reappraisal on cortisol recovery. However, trait reappraisal predicted faster cortisol recovery in healthy populations but not in the SAD group. These findings were discussed in terms of their difficulty in engaging in reappraisal effectively in the SAD group. In this respect, a strategy promoting cortisol recovery in populations with social anxiety needs additional examination. Further, the observation by Lewis et al. [16] is based on trait tendency to engage in emotion regulation strategies, and thus experimental examination to test whether engaging in an adaptive emotion regulation strategy instead of PEP promotes cortisol recovery is needed. One commonly used strategy that counteracts PEP is the strategy of distraction [17,18], which involves diverting attention away from an emotional situation and directing it toward independent neutral contents [19]. Engaging in distraction can reduce engagement in PEP, which may facilitate cortisol recovery. Considering that existing findings on the relationship between PEP and cortisol recovery are based on correlational study designs, examining this relationship with experimental manipulation of PEP would provide further support for this phenomenon. The effect of experimentally induced distraction on cortisol levels has been examined in previous studies. Zoccola et al. [20] compared cortisol recovery following psychosocial stress between experimentally induced rumination and distraction groups and discovered that distraction was associated with faster cortisol recovery. However, this finding is based on a healthy university student population, and no study has directly examined the effect of distraction on cortisol recovery among socially anxious individuals.

Thus, the aim of the present study was to experimentally test whether distraction (relative to PEP) following a social-evaluative stressor can promote cortisol recovery in individuals with subclinical social anxiety. We hypothesized that greater cortisol recovery would be observed for those who engaged in distraction compared with those who engaged in PEP following an acute stressor.

## 2 Method

### 2.1 Participants

Participants were recruited through advertisements posted around the university campus. On the advertisements, the requirement was noted that only those who tend to feel anxious in social interactions or performances in public would be eligible to participate. Applicants were individually invited to the laboratory for a detailed explanation of the study and screening. For the screening, applicants responded to the self-report version of the Liebowitz Social Anxiety Scale (LSAS) [21]. We utilized the Japanese translated version of the LSAS [22]. Those who showed willingness to participate and met the inclusion criteria participated in the experiment, which was conducted on a different day. Individuals were deemed ineligible if they met any of the following criteria: (a) a score below 30 on the LSAS (a cutoff score for classifying participants with social anxiety disorder with high sensitivity and specificity) [23]; (b) a history of a diagnosed psychiatric disorder; (c) stressful experiences immediately prior to entering the laboratory; (d) a history of smoking; (e) use of medications that could affect cortisol responses (e.g., oral contraceptives, β-blockers); (f) suffering from severe sleep disturbance or fatigue; and (g) irregular menstruation (for female participants). Female participants also provided menstrual phase information on the day of the experiment. To determine a sufficient sample size for examining our primary hypothesis (i.e., an effect of distraction cortisol recovery), we conducted a power analysis using G*power 3.1 [24]. We aimed to achieve 80% statistical power for a medium interaction effect (Cohen's f = 0.25, η^2^ ≈ 0.06) on two-way mixed ANOVAs to examine the effect of distraction induction on cortisol as well as subjective anxiety, which yielded 36 participants in total. This referenced effect size was almost comparable to the one observed in a previous study that examined the effect of distraction on cortisol (f^2^ = 0.07) [20]. To meet this sample size requirement, forty-two individuals completed all the tests. Participants were randomly allocated to either the distraction or the post-event processing (PEP) group. They were asked to refrain from vigorous exercise, alcohol, caffeine, and food for 1 h prior to study participation. All participants provided written informed consent and were told that they could withdraw from the study at any time. Participants were compensated for their participation with a book coupon worth 3000 Japanese yen. The study protocol was approved by Waseda University Academic Research Ethical Review Committee (approval number: 2016–276) and conducted in accordance with the Declaration of Helsinki.

### 2.2 Stress induction

We used a standard acute psychosocial stress test, namely the Trier Social Stress Test (TSST), that required participants to deliver a speech and perform mental arithmetic in front of a jury [25]. First, participants were led to a different room where they were introduced to a jury, consisting of a male and female observer wearing white lab coats. Second, they were then given 10 min to prepare for a mock job interview. Third, the participants underwent a 5-min mock job interview, where they were instructed to deliver a speech on why they would be an ideal job candidate in front of a jury and video camera. Fourth, after the interview participants performed a 5-min mental arithmetic task, in which they were asked to sequentially subtract 13 from 2083 as quickly and accurately as possible. When they made any error, feedback about the answer's accuracy was provided and they were requested to start over. The jury was trained to communicate with participants in an unresponsive, impassive manner and to maintain a stoic expression throughout the TSST.

### 2.3 Experimental manipulation

After completing the TSST procedure, participants were randomly assigned to one of two groups with stratification based on sex, according to a random numbers table that had been created before the data collection. No blinding procedures were performed. Those in the distraction group were asked to complete a distraction induction task [26]. To apply the distraction task (which was originally developed in English) to the Japanese population, we conducted a preliminary survey to choose appropriate items from the item pools in the original task, such that they were easy for Japanese participants to imagine. Ten volunteers rated the vividness of the images induced by the translated distraction items on a five-point scale (1: not vivid at all, 5: completely vivid and realistic). Of the original 45 items, we selected the top 16 items (the same as the number of items used in the PEP induction) that scored high on vividness (mean vividness = 3.41, SD = 0.47). Each item (e.g., “the shape of a large black umbrella”) was preceded by the phrase “Think about.” These procedures were intended for diverting attention away from the thoughts about the TSST and directing it toward independent neutral contents. Those assigned to the PEP group were asked to complete a guided rumination form [27], with 16 questions intended to elicit PEP (e.g., asked to list criticisms of their speech). All participants worked through the assigned task at their own pace for 10 min.

To confirm that this manipulation was successful, participants completed a manipulation check questionnaire. The questionnaire consisted of 5 items rated on a five-point scale (0 = not at all, 4 = very much). Four items were from Blackie and Kocovski [17], measuring the levels of PEP (3 items) and distraction (1 item) during the manipulation phase. Additionally, we added one item to assess thought suppression, which can be an inexplicit form of PEP, to confirm that participants were not engaged in it.

### 2.4 Measures

#### 2.4.1 Self-report measures

Demographic information and self-report data were collected during the adaptation phase of the laboratory visit. To assess social anxiety symptoms in more detail, the Social Phobia Scale (SPS) and the Social Interaction Anxiety Scale (SIAS) were utilized [28]. The SPS and the SIAS are self-report measures consisting of 20 items rated on a five-point Likert scale to assess anxiety in response to performing in public and social interaction, respectively (range: 0–80). Additionally, we utilized the Self-Beliefs related to Social Anxiety scale (SBSA) [29] to assess maladaptive cognitions associated with social anxiety. The SBSA is a self-report measure consisting of 15 items rated on an 11-point Likert scale to assess maladaptive beliefs related to social anxiety (range: 0–150). Further, the Center for Epidemiologic Studies Depression scale was utilized (CES-D) [30] to assess and control for depressive symptoms while examining cortisol recovery. The CES-D is a self-report measure consisting of 20 items rated on a four-point Likert scale to assess depressive symptoms (range: 0–60). We used the Japanese translated and validated versions of the SPS [31], SIAS [31], SBSA [32], and CES-D [33]. In addition to using these self-report measures, we assessed subjective state anxiety during the experiment using a Visual Analog Scale (VAS). Anchor values of zero and 100 were defined as “not at all” and “extremely” anxious, respectively.

#### 2.4.2 Post-stress thought sampling

After the experimental manipulation, all participants performed three rounds of the choice reaction time (CRT) task, to assess PEP in the post-manipulation period. This paradigm has been used routinely in previous studies that assess self-generated thoughts [34]. Indeed, this paradigm has been applied to assess self-generated thoughts after the TSST [15,35]. During the CRT task, participants observed sequences of white digits displayed on a black background of a computer screen while waiting for a red-colored digit to appear, at which point participants had to indicate the parity of this target (odd or even) with a button press. White digits were presented for 1000 ms, and colored stimuli were presented for 2000 ms. Events were separated by a fixation cross at a random duration (2,200, 2,800, 3,200, or 4400 ms). Targets (or question marks) and non-targets were presented at a ratio of approximately 1/6. During this task, PEP occurrences were recorded using the thought-probe method. During the task, participants were intermittently interrupted with questions, to which they had to respond “Yes” or “No” via a button press. Since PEP involves two aspects, namely thoughts about negative social events and cognitive interference [36], we used two questions representative of these components: “Were you just thinking about negative things that occurred during the interview task?” and “Were your thoughts about the interview task just interfering with your concentration?” These questions were developed based on the Post-Event Processing Questionnaire-Revised (PEPQ-R) [37]. In total, nine probes for these two questions were presented during the tasks. Individuals’ levels of PEP were defined as the number of “Yes” responses to the questions during this task (range: 0–18).

#### 2.4.3 Cortisol levels

Participants were asked to draw saliva from their mouths for 2 min and drool into a specimen tube through a 4-cm long straw (passive drool). Saliva samples were frozen in a freezer at temperatures below −20 °C until assay. Salivary cortisol levels were measured by means of enzyme-linked immunoassay using a commercial kit from Salimetrics (State College, PA, USA). The inter-assay coefficient of variation across all assays was 7.7%, and the intra-assay coefficient of variation was 4.6%. All cortisol values were Box-Cox power transformed to normalize their distribution using the following formula [38]:

X’ = (X^0.26^ − 1)/0.26

### 2.5 Procedure

**Fig. 1:**
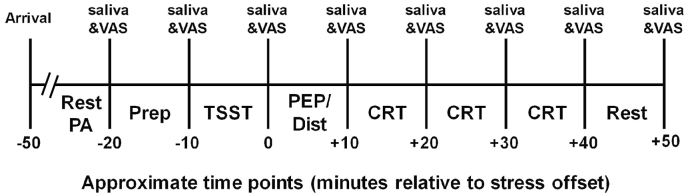
Overview of the testing timeline. PA = psychological assessments, Prep = preparation time for the speech task, TSST = Trier Social Stress Test, PEP = post-event processing induction, Dist = distraction induction, CRT = Choice reaction task.

All tests were performed in the afternoon (between 1300 and 1830) to control for circadian variation in cortisol activity. Fig. 1 shows an outline of the whole experimental procedure. At the beginning of the experiment, participants provided written informed consent. To minimize the predictability of the experimental manipulation, participants were just informed that the purpose of the study was “to reveal the mechanism underlying prolonged physiological responses”; they were not informed that they were to be allocated to one of the two conditions for experimental manipulation, until the end of the study. Next, participants completed psychological assessment questionnaires, which took approximately 10 min. Participants then remained seated in a quiet room for 10 min to control for any potential confounders prior to initial cortisol sampling. Subsequently, participants completed the TSST. Following the TSST, participants were assigned 10 min to complete the assigned experimental manipulation task and thereafter completed the manipulation check questionnaire. Then, all participants completed three rounds of CRT, during which PEP levels were assessed through the thought-sampling procedure. Finally, participants were given an additional 10 min to rest. Throughout the testing period, participants had to refrain from eating and drinking anything, except for having some water. Saliva collection and assessment of state anxiety were conducted at eight time points: baseline, after speech preparation, just after the TSST, after experimental manipulation, after each block of the CRT, and after a 10-min rest following the cognitive tests. These assessments largely corresponded to the following time periods with respect to the TSST offset: −20, −10, 0, +10, +20, +30, +40, and +50 min.

### 2.6 Data analysis

For data reduction, two participants were excluded from the final analyses; one with irregular menstruation who was incorrectly deemed eligible and one with an extreme cortisol value (i.e., above 3*SD*s from the mean) even after power transformation. To ensure that the TSST successfully served as a social-evaluative stressor, we conducted a linear mixed model with time for subjective anxiety, with random intercepts for each participant. For manipulation check, we conducted a multivariate analysis of variance (MANOVA) with group for manipulation check items regarding PEP and distraction. Additionally, we conducted a linear mixed model with condition and CRT blocks (block 1 to 3) on PEP assessed using the thought sampling procedure. Furthermore, we conducted a linear mixed model with condition and time for subjective anxiety to test the effect of distraction on subjective anxiety.

To test our hypothesis regarding the effect of distraction versus PEP, we applied a two-piece multilevel growth curve modeling with landmark registration (GCM-LR) [39]. This approach enables simultaneous modeling of the cortisol activation to the peak, the absolute peak value, and the cortisol recovery from the peak, while controlling for the individual peak timing in response to the acute stress. This approach involves 3 steps (see Ref. [39] for detail).

First, individuals’ post stress peaks were identified according to the peak identification procedure proposed by Lopez-Duran et al. [39]. To this end, individual cortisol values were inspected, and peaks were defined as the first measurement point that was at least 15.5% greater than the baseline value and was followed by a decline or a plateau. This 15.5% cut-off criterion has been shown to effectively distinguish between cortisol responders and non-responders [40]. Participants with peaks were labeled as “responders”, otherwise were labeled “non-responders”.

Following the peak identification, a new variable reflecting the minutes from the peak using the following formula:

MinFromPeak = (PeakTime − Time) × −1.

For those without peaks (i.e., non-responders), the mode peak time among responders (+10 min from the TSST offset) was used to model their responses.

Finally, we created two variables to represent minutes before (TimeBeforePeak) and after the peak (TimeAfterPeak) using the following formulas:

IF MinfromPeak <0 then TimeBeforePeak = MinfromPeak

Else MinfromPeak = 0.

IF MinfromPeak >0 then TimeAfterPeak = MinfromPeak

Else MinfromPeak = 0.

Using these variables, we conducted a multilevel random effect model predicting the cortisol trajectories in response to TSST. The unconditional fixed effects model was defined as:

Cortisol = β_0_ + (β_1_ × TimeBeforePeak) + (β_2_ × TimeAfterPeak) + e

where β_0_ is the intercept (peak), β_1_ is the activation slope, and β_2_ is the recovery slope. In the conditional model, a contrast-coded dummy variable reflecting conditions (−1: PEP condition, 1: Distraction condition) was included. Additionally, to control for the potential variations in cortisol activation and recovery due to baseline variables, sex, body mass index (BMI), depression (CES-D total score), and social anxiety (composite score of SPS and SIAS total score; see Ref. [15]) were included as covariates.^1^ All models included random intercepts and slopes in addition to the fixed effects of activation and recovery slope, while controlling for cortisol baseline levels. Finally, as a supplementary analysis, we calculated cross-correlation coefficients for state anxiety and cortisol levels to assess the covariation between subjective anxiety and cortisol changes [41]. All analyses were conducted in R 4.1.2. The significance levels were set at 0.05 (two-tailed).

## 3 Results

### 3.1 Preliminary analysis

The overall cortisol response rate to the TSST (showing increase) was 62.5%, which was a bit lower but almost comparable to the response rates in previous studies (>70.0%) [25]. Of these, most participants had their cortisol peak at the +10 min measurement point (72.0% in cortisol responders). Descriptive statistics for demographic information and self-report questionnaires in the distraction group and PEP group are shown in Table 1. There were no significant differences in age, sex ratio, BMI, and self-report questionnaire scores (LSAS, SPS, SIAS, CES-D, and SBSA), confirming homogeneity between the groups.

**Table 1:** Group means (±SD) for demographics and questionnaires scores.

	Distraction	PEP	*t*/χ^2^	*p*
Total *n*	19	21	-	-
Female: Male	11:8	13:8	0.00	1.00
Age	21.05 (1.87)	21.14 (2.59)	−0.13	.90
BMI	20.39 (2.05)	20.20 (2.51)	0.26	.80
SPS	19.74 (9.85)	18.48 (14.72)	0.32	.75
SIAS	33.74 (7.33)	33.48 (9.02)	0.10	.92
SBSA-CB	23.21 (13.53)	27.38 (16.16)	−0.89	.38
SBSA-UCB	15.68 (7.48)	15.19 (8.61)	0.19	.85
SBSA-HS	16.68 (9.02)	18.62 (10.65)	−0.62	.54
CES-D	14.47 (8.59)	12.81 (7.74)	0.64	.53
LSAS	58.05 (14.74)	50.62 (18.95)	1.39	.17

SPS = Social Phobia Scale; SIAS = Social Interaction Anxiety Scale; SBSA = Self-Beliefs Related to Social Anxiety Scale; CB = conditional beliefs; UCB = unconditional beliefs; HS = high standard beliefs; CES-D = Center for Epidemiologic Studies Depression scale; LSAS = Liebowitz Social Anxiety Scale.

### 3.2 Subjective anxiety response to the TSST

For subjective anxiety, there was a significant effect of time (*F* [7, 273] = 73.10, *p* < .01). Multiple comparisons using Sidak correction revealed that participants exhibited elevated anxiety in anticipation of the TSST (at the 10 min time point; *p* < .01), which lasted even after they completed the TSST (at 0 min time point; *p* < .01).

### 3.3 Manipulation check

A MANOVA examining the group difference in the 5 manipulation check items was conducted. As a significant multivariate effect of group was observed (Pillai's trace = 0.50, *p* < .01), post hoc analyses with Games-Howell method were performed to further assess these differences. Means and SDs are shown in Table 2. The PEP group exhibited higher scores on items assessing PEP-related thoughts (items 1, 3, and 4). Additionally, the distraction group exhibited higher scores on item 2, which reflects distraction-related thoughts. There was no group difference on item 5, which reflects thought suppression.

**Table 2:** Comparison on manipulation check items between groups.

Items	Distraction		PEP		*t*	Cohen's *d*	*p*
	Mean	SD	Mean	SD			
1. How much did you think about the speech and arithmetic tasks?	2.00	0.94	3.33	1.06	−4.20	1.32	<.01
2. How distracted were you from thinking about the speech and arithmetic tasks?	2.05	0.97	0.62	0.67	5.39	1.73	<.01
3. How much did you dwell on the speech and arithmetic tasks?	1.42	0.84	2.19	1.40	−2.13	0.65	.04
4. Did you experience distressing thoughts about the speech and arithmetic tasks?	1.26	0.99	2.33	1.06	−3.29	1.04	<.01
5. Did you attempt to suppress distressing thoughts about the speech and arithmetic tasks?	0.68	0.95	0.76	0.83	−0.28	0.09	.79

We also performed a linear mixed model for PEP scores, assessed through thought sampling, to examine whether the effect of distraction induction persisted at later time points. There was a significant effect of CRT block (*F* [1, 78] = 5.87, *p* = .02), indicating a reduction in PEP occurrence over time regardless of group. However, there was no significant interaction between condition and CRT block (*F* [1, 78] = 0.06, *p* = .81).

**Fig. 2:**
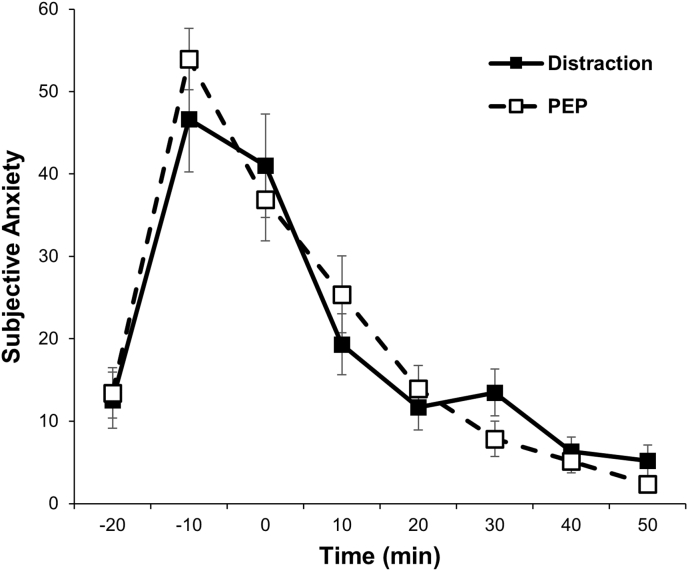
Subjective anxiety trajectories following the TSST in each group.

To examine the effect of distraction on subjective anxiety, we performed a linear mixed model examining the effect of distraction induction on subjective anxiety across time. While the main effect of time was significant (*F* [7, 266] = 63.53, *p* < .01), the condition × time interaction was not significant (*F* [7, 266] = 1.26, *p* = .27), suggesting no difference regarding changes in subjective anxiety between the conditions. The trajectories of subjective anxiety in each group are shown in Fig. 2.

### 3.4 Unconditional model predicting cortisol trajectory

To confirm whether the expected increase and decrease in cortisol levels occurred following the TSST, we examined the unconditional model of cortisol trajectory, where no level-2 predictors were included in the model. This unconditional model showed that salivary cortisol levels significantly increased from the baseline (*b* = 0.013, *t* = 4.61, *p* < .01) and decreased from the peak (*b* = −0.009, *t* = −9.45, *p* < .01). Thus, the expected increase and decrease in cortisol levels were observed. Given this result, we further examined a conditional model that examined the effect of experimental manipulation.

### 3.5 Conditional model predicting cortisol trajectory

**Fig. 3:**
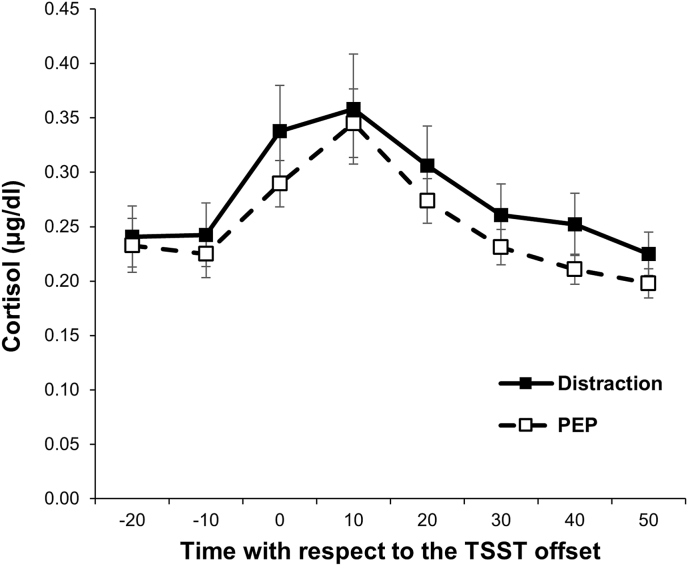
Cortisol response trajectories following the TSST in each group.

We conducted a conditional model to examine the predictive effect of experimental manipulation on cortisol recovery. The model summary is provided in Table 3. The effect of condition on cortisol recovery, as indicated by the interaction of condition and recovery slope, was not significant (*t* = 1.95, *p* = .06). Social anxiety as a covariate was the only significant predictor of cortisol trajectory, which predicted slower activation, lower peak, and slower recovery. Cortisol values in each group are shown in Fig. 3.

**Table 3:** Model summary predicting salivary cortisol peak, activation, and recovery.

	*b*	*SE*	*t*	*p*
(Intercept)	−0.980	0.050	−19.44	< .01
Baseline	0.120	0.060	2.00	.05
Sex	0.050	0.060	0.84	.41
BMI	0.105	0.066	1.58	.12
Social anxiety	−0.173	0.068	−2.56	.02
Depression	0.096	0.073	1.32	.20
Condition	−0.009	0.051	−0.17	.87
Activation slope	0.012	0.002	5.70	< .01
Baseline × Activation slope	−0.007	0.003	−2.68	.01
Sex × Activation slope	0.004	0.003	1.38	.18
BMI × Activation slope	0.001	0.003	0.52	.61
Social anxiety × Activation slope	−0.009	0.003	−3.20	< .01
Depression × Activation slope	0.005	0.003	1.47	.15
Condition × Activation slope	−0.001	0.002	−0.56	.58
Recovery slope	−0.009	0.001	−10.39	< .01
Baseline × Recovery slope	−0.001	0.001	−0.61	.54
Sex × Recovery slope	−0.002	0.001	−1.52	.14
BMI × Recovery slope	−0.002	0.001	−1.41	.17
Social anxiety × Recovery slope	0.003	0.001	2.22	.03
Depression × Recovery slope	−0.001	0.001	−1.08	.29
Condition × Recovery slope	0.002	0.001	1.95	.06

### 3.6 Covariance between subjective anxiety and cortisol changes

**Fig. 4:**
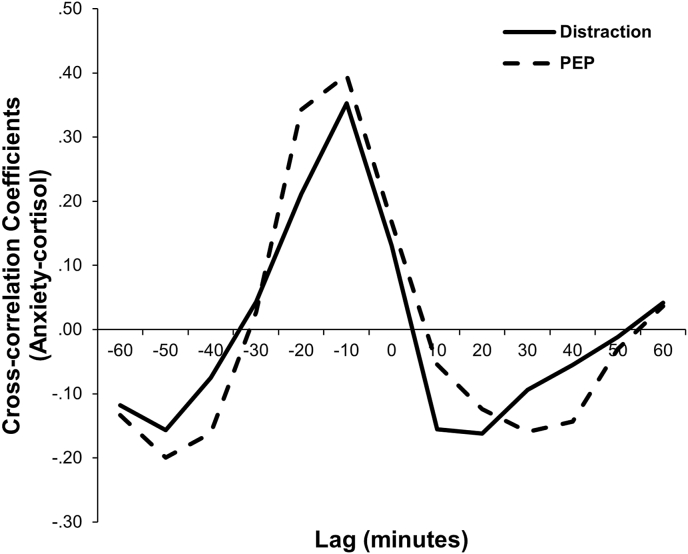
Cross-correlation coefficients between subjective anxiety and cortisol changes. A negative lag indicates that subjective anxiety precedes cortisol level changes. For example, cross-correlation at lag –10 min shows the average association of all possible pairs of subjective anxiety with cortisol 10 min later.

Fig. 4 shows the cross-correlation coefficients between subjective anxiety and cortisol changes. In both conditions, cross-correlation showed the highest values with a –10-min lag (Distraction condition: *r* = 0.35; PEP condition: *r* = 0.40). This indicates subjective anxiety and cortisol co-vary in response to the TSST and that subjective anxiety precedes cortisol changes.

## 4 Discussion

The current study aimed to examine whether distraction facilitates cortisol recovery following a social-evaluative stressor in individuals with subclinical social anxiety. By experimentally manipulating distraction after the stressor (induced distraction vs. PEP), we were able to examine its effect on cortisol recovery. Contrary to the hypothesis, no clear effect of distraction on cortisol recovery was observed.

Our hypothesis that those who engaged in distraction would show greater cortisol recovery than those who engaged in PEP following an acute stressor was not supported. This suggests that our distraction procedure was not effective enough to reduce PEP in the present experimental setting. The fact that PEP levels did not differ in the post-manipulation period, assessed through thought sampling, further supports this interpretation. This null effect of distraction can be understood in terms of the intensity of the social stressor utilized in this study. For a social stressor, we utilized a well-validated stress testing protocol (the TSST), which comprises a mock-job interview and mental arithmetic for 10 min with explicit negative performance feedback. In contrast, prior studies reporting reduction in PEP by distraction mostly use social stressors with less intensity (e.g., an impromptu 5-min speech) [17,20]. Explicit negative performance feedback can cause longer-lasting PEP [42], and, thus, it is possible that it was relatively difficult for participants to cope with PEP using distraction in our study setting. It would be worthwhile to examine the effect of a more extensive distraction procedure such as one lasting a longer time.

At the same time, we should also consider the possibility that PEP can be a kind of “coping mechanism” for individuals with elevated social anxiety. Although not significant, there was a trend toward an interaction between condition and recovery slope, suggesting that PEP induction, rather than distraction induction, is associated with faster cortisol recovery. This observation may be consistent with the previous finding that self-generated PEP is associated with faster cortisol recovery for those with high levels of social anxiety [15]. Some individuals with SAD are reported to experience a sense of relief when engaging in PEP as they believe they can improve themselves by doing so [43]. This sense of relief can be associated with faster cortisol recovery, which may explain the lack of significant difference in cortisol recovery between distraction and PEP conditions. However, even if this speculation is true, it does not mean that PEP can be an effective emotion regulation strategy after social evaluative situations in the long term. Individuals with severe social anxiety may ruminate for hours or even days over their perceived social failures following social encounters [44]. In this sense, our observation is limited to only a short period after exposure to an anxiety-provoking situation, which makes it difficult to conclude whether PEP can be an effective coping mechanism for promoting cortisol recovery. To further elucidate the role of PEP and cortisol response in individuals with SAD, it would be useful to utilize an experience sampling approach and saliva sampling throughout daily routines [45]. This type of investigation would also be important from the perspective of the generalizability of our findings, considering that the degree of off-task thinking does not clearly transfer from the laboratory to daily life, and that the relationship between thought content and cortisol levels also differs between the laboratory environment and daily life [46].

Although we examined the effect of distraction, future examination of different coping mechanisms and cortisol recovery would be worthwhile. Distraction can be considered a passive kind of coping mechanism since it does not focus on directly confronting the negative thoughts and feelings. By contrast, an active kind of coping mechanism that involves confrontation with the negative thoughts and feelings can also be effective in reducing PEP, thereby promoting cortisol recovery. One plausible approach is self-compassion, which includes being open to one's own suffering, experiencing feelings of care and kindness toward oneself, taking a non-judgmental attitude toward one's inadequacies and failures, and recognizing that one's own experience is part of the common human experience [47]. There are some findings suggesting the relative effectiveness of self-compassion compared to distraction in response to a negative mood induction [48] and the effectiveness of self-compassion in reducing PEP [49].

Several limitations of the present study should be noted. First, we did not include a passive control group, which limits the interpretation of the findings. Inclusion of the passive control group allows us to examine the effect of distraction or PEP in comparison to “natural” coping with the social evaluative situation. Regarding the experimental manipulation, assessment of the predictability of the experimental manipulation and manipulation checks not relying on self-reported measures may further increase the validity of the manipulation. Second, while we selected participants who exhibited at least elevated levels of social anxiety, we did not utilize a “gold-standard” such as the structured clinical interview. Third, we did not control for the menstrual cycle of female participants, which could affect cortisol responsivity [50]. At the same time, a recent study recommends statistically controlling for oral contraceptive use rather than the exclusion of oral contraceptive users, considering the high proportion of females taking oral contraceptives [51]. More rigorous control for these possible confounders is desired. To further control for the possible confounder associated with cortisol reactivity, prior experience with the same stress protocol should also be considered among the exclusion criteria, as repeated exposure to the same stressor causes habituation [52]. Finally, the sample size in our study was relatively small and a replication with a larger sample is desirable. Although our sample size was based on a-priori power analysis to detect the “medium” size effect for distraction, more conservative assumptions about effect sizes may have been appropriate when examining the effect of distraction in individuals with social anxiety.

Notwithstanding these limitations, our study examined the effect of experimental manipulation of distraction on cortisol recovery in individuals with social anxiety for the first time. Future studies should attempt to refine the procedures for distraction and examine their effect for better cortisol recovery. At the same time, it would be valuable to investigate more suitable approaches than distraction based on the clinical features of social anxiety.

## Funding

This work was supported by Japan Society for Promotion of Science KAKENHI (grant number 16J10256).

## CRediT authorship contribution statement

**Shunta Maeda:** Funding acquisition, Conceptualization, Methodology, Formal analysis, Investigation, Writing – original draft. **Chihiro Moriishi:** Methodology, Investigation, Writing – review & editing. **Hiroyoshi Ogishima:** Methodology, Investigation, Writing – review & editing. **Hironori Shimada:** Supervision, Writing – review & editing.

## Conflict of interest

Shunta Maeda, Chihiro Moriishi, Hiroyoshi Ogishima, and Hironori Shimada declare that they have no conflict of interest.

## Footnotes

1 We included the composite score of SPS and SIAS total score for social anxiety instead of the LSAS score. This was because the SPS and SIAS scores could reflect the levels of social anxiety on the day of the experiment; the LSAS was assessed on a different day prior to the experiment. We also intended to keep consistency with the relevant study for social anxiety measure [15].
